# Implementing a primary care disease management concept for venous leg ulceration: findings of a mixed-methods process evaluation in the Ulcus Cruris Care trial

**DOI:** 10.1186/s12913-026-14674-0

**Published:** 2026-05-07

**Authors:** Regina Poß-Doering, Thomas Fleischhauer, Nina Sander, Gunter Laux, Michel Wensing, Joachim Szecsenyi, Jonas. D. Senft

**Affiliations:** 1 Medical Faculty, University Heidelberg, Heidelberg, Germany; 2 Department of Primary Care and Health Services Research, University Hospital Heidelberg, Heidelberg, Germany; 3 aQua Institute, Göttingen, Germany

**Keywords:** Chronic wounds, Venous leg ulcer, case management, general practice, Education

## Abstract

**Background:**

Care analyses show that evidence-based measures such as compression therapy and promotion of mobility are rarely implemented in treatment of Venous leg ulcers. The Ulcus Cruris Care project developed a disease- management approach to support evidence-based venous leg ulcer treatment in general practices. This included online training and three e-learning modules for practice teams, software-supported case management, involvement of non-physician assistants, and promotion of patient activation and education. The intervention program was implemented within a multicenter randomized controlled trial (Ulcus Cruris Care). A mixed-methods process evaluation explored intervention fidelity and perceived effects to identify improvement potential.

**Methods:**

The cross-sectional process evaluation design applied semi-structured guide-based qualitative telephone interviews and a study-specific survey to evaluate the implementation process of the program.

**Results:**

*N* = 38 survey questionnaires were completed and *n* = 27 interviews were conducted (*n* = 10 general practitioners, *n* = 10 non-physician assistants, *n* = 7 patients). Findings indicate high intervention fidelity regarding completion of the online training (100%), the e-learning modules (between 61% and 48%), application of standard operating procedures (100%), patient education material (91%), and case management software (91%). Practice teams and patients positively perceived the role of non-physician assistants as case managers and their involvement in wound treatment and patient education. Overall, the program was perceived as effective in promoting change in treatment routines, particularly the regular use of compression therapy as the most effective treatment measure, improving patient and practice team education, and ultimately, wound healing.

**Discussion:**

The intervention program was expected to increase the use of compression therapy, speed up healing, and reduce the use of medical resources. Participants in this process evaluation perceived the program as supporting structured venous leg ulcer treatment, increasing relevant knowledge among practice teams and patients, and encouraging more active involvement of patients and relatives. The outcome analysis in the Ulcus Cruris Care trial strengthened these findings and suggested a potential benefit of the intervention.

**Conclusion:**

Promotion of comprehensive VLU treatment and care in general practices, including a regular use of compression therapy and active patient participation as facilitated by this intervention program appears to be largely suitable for a venous leg ulcer case management approach.

**Trial registration:**

The Ulcus Cruris Care trial protocol was registered in the German Clinical Trials Register on August 30, 2021 (DRKS00026126).

**Supplementary Information:**

The online version contains supplementary material available at 10.1186/s12913-026-14674-0.

**Table:** 

Text box 1. Contributions to the literature
• Application of chronic wound care concepts still needs encouragement in primary care in Germany. Implementation of an evidence-based care concept that includes compression therapy can promote faster wound healing and improve quality of life.
• In our multi-faceted program, implementation strategy clusters followed expert recommendations to foster change and train and support care providers and patients alike.
• Evaluation of the implementation process facilitated insights regarding intervention fidelity and perceived effects and supports assessment of this program as suitable for a broader disease management approach.

## Background

Chronic wounds are considered a worldwide problem. It is estimated that in developed countries about 1–2% of the population will experience a chronic wound during their lifetime. In low income countries, etiologies may be different, yet there is a common denominator which is “impaired healing” [1]. Venous leg ulcers (VLU) account for about 70% of chronic lower extremity ulcers and about 1% of the population and 3% of people over 80 years of age are presumed to suffer from VLU [2, 3]. Due to aging populations, a steadily growing prevalence can be expected [4]. Thus, lower-extremity ulcerations are very common and have a major impact on public health [5].

In Germany, about 1.8 million people are affected by VLU [6], yet standardized pathways for their treatment have hardly been established, despite European guidance being available [7, 8]. Thus, affected patients may suffer chronification, morbidity and considerable impairment of quality of life [9, 10]. Compression therapy carried out in accordance with existing guidelines counteracts the venous disease and thus the cause of the wound healing disorder and has a proven effect on wound healing. A recent study in Germany concluded that in VLU outpatient care, approximately 80% of patients receive neither consistent compression therapy nor surgical treatment and are therefore inadequately treated [11]. Though more recent studies are necessary as confirmation, compression therapy can still considered to be implemented in less than 50% of cases and often incorrectly applied [12, 13]. Other effective treatment elements such as promoting mobility and exercising are also rarely addressed [14]. In contrast, modern wound therapeutics (i.e. dressings) are often used for topical therapy, even though there is no objective evidence of beneficial effects [15]. Reasons for wound care deficits are assumed to be ranging from insufficient care provider knowledge regarding compression therapy and its practical application [16, 17], to a lack of standardized outpatient care concepts, increasing time pressure in practices [18], and a passive, uninformed patient role during treatment [19].

In Germany, care providers such as general practitoners (GPs) and their teams, outreach nursing services and certified wound managers (non-physician medical assistants with wound-specific additional training) can be involved in VLU treatment and be the primary care providers for patients with VLU. However, there is still a knowledge gap, in particular regarding the application and practical implementation of compression therapy [20]. The project Ulcus Cruris Care (UCC) aims to establish a patient-oriented and evidence-based care concept for VLU treatment in general practice by ensuring care coordination and multidisciplinary treatment. Educational interventions were developed to specifically enable GPs and their non-physician medical assistants (MA) to fill a central role in VLU management and support patients and relatives in taking an active role in the treatment process. Also, e-learning and computerized case management were integrated. As nursing staff are rarely employed in German general practices, their skills and expertise in wound management are mostly available in outreach nursing services and thus not integrated in general practice care. MA in Germany are comparable to MA in USA regarding education, tasks, and remuneration [21], but have different qualifications than nursing staff.

The UCC intervention aimed to address this issue by expanding the capabilities of primary care teams, - particularly non-physician healthcare professionals - with regard to the practical implementation of compression therapy as a clinical skill, and, importantly to monitoring its effects. The study hypothesized that implementation of the concept would generally promote evidence-based treatment and the use of compression therapy, potentially leading to faster wound healing, improved quality of life for affected patients and reduced use of medical resources. After piloting of the intervention program [22], a multicenter cluster-randomized controlled study (RCT) was conducted in general practices [23]. A process evaluation accompanied the implementation process to investigate intervention fidelity, perceived effects and participant satisfaction with the intervention, and opportunities for improving the intervention components [24].

## Methods

### The UCC intervention

The UCC intervention was developed to improve primary care treatment of VLU. Following a status quo survey [25], a multifaceted intervention for VLU outpatient treatment was developed, successfully piloted [22], and subsequently implemented as care concept in *n* = 44 general practices in Germany for a total of *n* = 53 patients with VLU within a multicenter randomized controlled trial [23]. The intervention addresses implementation strategy clusters suggested by the Expert Recommendations for Implementing Change (ERIC) taxonomy [26, 27] and comprises five main components:

(1) On-demand online training and e-learning courses for GPs and MAs (cluster: Train and Educate Stakeholders); (2) Standard operating procedures (SOP) for evidence-based VLU treatment (cluster: Support Clinicians); (3) Software-supported case management (cluster: Change Infrastructure); (4) Strong involvement of MAs in case management, wound care, and patient education (cluster: Support clinicians; Develop Stakeholder Interrelationships); (5) Printed educational material and e-learning course for patients (cluster: Train and Educate Stakeholders; Engage consumers).

The online training and e-learning courses were based on relevant literature and guidelines [14, 28–35] and focused on VLU pathophysiology and chronic venous insufficiency, effectiveness and practical aspects of evidence-based compression therapy, local wound treatment, and patient education. A previous publication describes the implementation program in detail [24].

### Study design

As a part of the UCC trial [24], an embedded mixed-methods process evaluation was conducted to assess and evaluate the implementation process of the UCC intervention regarding intervention fidelity, perceived effects and participant satisfaction with the intervention, and to identify improvement potential. The convergent mixed-methods approach [36] comprised semi-structured guide-based qualitative telephone interviews and an accompanying survey study. This process evaluation addressed all participant groups in the intervention arm of the trial to gain insights into their experiences and perceptions regarding the intervention. In process evaluation, assessment of fidelity, perceived effects, and potentially necessary adaptation can be informed by exploration of applicability and acceptability of an intervention within the respective setting [37, 38]. To support the appraisal of the UCC intervention and avoid incorrect conclusions, this process evaluation also focused on the assessment of participant satisfaction (contentedness) with the intervention which is considered to provide useful feedback regarding potential improvements [39, 40].

### Recruitment

An information letter was sent by mail to approximately 800 regional GP practices collaborating with the study center inviting them to participate in the randomized controlled UCC trial. Interested practices were screened for eligibility by the study team. Inclusion criteria were to meet IT requirements for using the software-supported case management and at least one MA to be routinely involved in chronic wound treatment and care. The intervention explicitly targeted GP practices that were not specialized in wound care and treated about six VLU patients per year. Therefore, GP practices with more than 20 VLU patients per year could not participate. A pragmatic convenience sampling strategy was used for this process evaluation [41].

To be eligible for inclusion in the process evaluation, GPs, MAs, and patients had to be adult participants in the RCT intervention group, with good mastery of German. Patients also had to have a diagnosed VLU that had been present for a maximum of 12 months. MAs had to be actively involved in wound care. All patients in the intervention group were invited to participate in the process evaluation by the intervention group practices and received printed information about the process evaluation and its objectives, and a consent form. If written consent was given, contact details (telephone number and address) were forwarded to the study team. All GPs and MAs from the intervention practices were contacted via telephone by the study team to invite them for participation in the process evaluation.

After completing the online practice training and treating at least two study patients, GPs and MAs who were willing to participate received a separate informed consent form. All participants were informed verbally and in writing about content and objectives of the study and gave written consent prior to participating in the process evaluation. Participants could decide whether they participated in the survey, an interview, or in both. Recruitment target was to include 10 GPs, 10 MAs, and 10 patients in this process evaluation. A small expense reimbursement of 50€ was offered for participation in an interview and an additional 20€ for filling in the study-specific questionnaires.

Integrated into the study protocol of the multicenter RCT [24], this process evaluation and its data collection instruments received a positive vote from the Heidelberg Ethics Committee (S-608/2021) and the Ethics Committee of the German Medical Association (B-F-2021-101).

### Data collection

For this process evaluation, study-specific self-reported survey questionnaires (Additional file 1) were developed to cover items regarding intervention fidelity (7 items), perceived (9 items) as well as non-anticipated effects (2 items), and improvement potential (15 items). Response options were provided on a 5-point Likert scale (rating: 1 = totally disagree; 5 = totally agree), and an optional free text field. A socio-demographic questionnaire was used to collect participant characteristics. The qualitative study used a semi-structured interview guide (see Additional file 2) to explore participant perceptions regarding acceptance of the intervention program, intervention fidelity, and perceived effects. Against the background of questions used in the evaluation of the pilot study [22], the interprofessional research team (Health Services Research, General practice, Clinical Research) developed interview guides for the three groups (GPs, MAs, patients) in an iterative process by collecting, discussing and subsuming appropriate questions and wording [42]. As per study protocol [24], the updated Consolidated Framework for Implementation Research [43] - an implementation determinant framework [44] designed to describe barriers and facilitators to implementation outcomes [45] - with its’ Implementation Process domain served as overall guidance for data collection, particularly regarding the constructs Teaming, Engaging, Reflecting & Evaluating and Adapting.

All interviews were conducted by junior and senior research team members (NS (f), TF (m)), audio-recorded, pseudonymized and transcribed via the software f4. Interviews were conducted at the workplace or the home office, participants were at home or at their workplace and knew interviewers through study-related conversations. No non-participants were present, no field notes were taken, and transcripts were not returned to participants. Data were organized and managed in MAXQDA Version 2022 (Release 22.7.0; Verbi Software) and stored on secure servers at the Department of Primary Care and Health Services Research, University Hospital Heidelberg, Germany.

### Data analysis

Quantitative and qualitative data were analyzed separately and then integrated to identify common concepts across both data sources. All quantitative and socio-demographic data were analyzed descriptively by three researchers (T.F, R.P.-D., N.S.) using Microsoft Excel software (Version 1808). Means, medians (med), standard deviations, maximum and minimum values, and absolute and relative frequencies were calculated. Verbal Likert scales were numerically recoded from 1 to 5 (totally disagree = 1, somewhat disagree = 2, partially agree = 3, somewhat agree = 4, totally agree = 5). For intervention fidelity, absolute and relative frequencies were calculated. The qualitative data were analyzed inductively using the Framework analysis method [46, 47] as a systematic approach to structuring data and enabling cross-category comparisons to identify key messages and potential contrasts [46]. All interview data were analyzed by a junior (Clinical Research) and a senior researcher (Health Services Research). Coding was discussed and reflected in regular meetings in the study team (R.P.-D., J.D.S, N.S.) and approximated if divergences occurred to reach inter-coder agreement. Information saturation was assessed when the identified range of new issues (code saturation) led to full understanding of these issues (meaning saturation) [48].

## Results

All process evaluation data were collected in the intervention group (*n* = 20 practices) during the UCC intervention phase between May 2023 and February 2024. A total of *n* = 27 interviews were conducted (*n* = 10 GPs, *n* = 10 MAs, *n* = 7 patients), *n* = 38 completed survey (*n* = 12 GPs, *n* = 15 MAs, *n* = 11 patients) and *n* = 30 socio-demographic questionnaires (*n* = 10 GPs, *n* = 11 MAs, *n* = 9 patients) were returned. Interview and survey participants were not necessarily the same individuals. Interview duration was between 08:31 min and 54:27 min (mean duration: GP = 20:16 min, MA = 23:21 min, patients = 19:18 min; total = 20:58 min). No practice actively declined to participate in this process evaluation. Table 1 describes the socio-demographic characteristics of the sample.

**Table 1:** Socio-demographic sample characteristics

	General practitioners *n* = 10	Medical assistants *n* = 11	Patients *n* = 9
Age mean (range)	54.5 (35–65)	48.1 (30–58)	76.8 (56–85)
Sex f (n)	4	11	6
Professional experience in years (range)	25.3 (6–37)	25 (09–35)	
Specialist in general practice (n) Specialist in internal medicine (n)	8 2		
specialized assistance in general practice (n)		8	
Working full-time (n)	10	4	1
Working in single practice (n)	6	6	
Working in rural area (n)	8	8	
Living in rural area (n)			6

The majority of patients were covered by statutory health insurance (*n* = 8), were no longer employed (*n* = 8) and almost half of them lived with a partner or relative (*n* = 4). All GPs stated that they were responsible for diagnosing, determining therapy, counseling and educating VLU patients and that they either engaged in wound care themselves, delegated it to an MA or carried it out as a team. MAs stated that they were involved in both, patient education and wound care and were responsible for applying compression bandages. Most of the practice teams described working regularly with nursing services when caring for VLU patients outside of the study.

The results reported below refer to participant ratings provided in the survey and stated participant perceptions from the qualitative data. Included quotes have been translated from German to English with due diligence and are presented with an indication of participant group (GP=general practitioner; MA = non-physician assistant; PAT=patient) and number of the interview. Findings from the survey are presented descriptively and complementary to the qualitative data. All findings are presented with regard to the intervention components along the evaluation target criteria: (1) Intervention fidelity, (2) Perceived effects, and (3) Improvement potential.

### Intervention fidelity

#### Practice team perceptions

All practice teams participating in the process evaluation attended and completed the online training (100%). The three e-learning modules were completed by *n* = 17 (58.6%), *n* = 15 (51.7%), and *n* = 14 (48.3%) GPs, and *n* = 22 (61.1%), *n* = 20 (54%), and *n* = 19 (51.3%) MAs in the intervention group.

Participating GPs indicated in the survey that they used the provided standard operating procedures (*n* = 12/12), the case management software (*n* = 11/12), the patient education material (*n* = 11/12), and informed about the optional patient e-learning (*n* = 7/12). Two GPs indicated they made all their participating patients aware of this option. Limitations were indicated regarding the patient e-learning in connection to patient age. GPs stated they saw relevance and benefits for the practice team and patient care in completing the online training (webinar), using the standard operating procedures (med 4.5 out of 5 each), the e-learning modules (med 4.25 out of 5), the software-supported patient monitoring and the printed patient education material (med 4 out of 5). One GP commented in a free text field that practice routines might need adaptation to fully make use of all intervention components.

In the interview study, all GPs (*n* = 12) and MAs (*n* = 11) reported they took part in the synchronous online training and completed the e-learning. In the online training format, exchange with other practices and the inclusion of interesting case studies were highly appreciated. Regarding content, participants in both groups ranked compression therapy and local wound treatment as particularly relevant. GPs also rated the content on patient education as particularly relevant.

MAs and GPs also stated that they found the SOP for guideline-compliant VLU care supportive and used it because “it was finally documented and clearly stratified […]” (GP07). One MA and one GP each perceived the SOPs as rather unhelpful. Two practices reported that they had laminated the SOPs for quick and easy use and hung them up clearly visible in the treatment room. Some participants passed on the SOPs to nursing services and social care units so they could also benefit from the knowledge.

*[…] I have now also printed this out with these [SOP]*,* […]*,* we now have this as a guide*,* the others are now also following it. (GP08)*

All GPs and almost all MAs reported using the written patient information, handing it out to patients and also passing it on to nursing and social services for their education. Both professional groups shared the view that the structured verbal education together with dissemination of written information was sustainable and effective. GPs stated that they informed patients about the possibility of making use of the on-demand e-learning, yet patients did not engage with it. According to the interviewees and free text fields in the survey questionnaires, this was assumed to be due to advanced patient age, lacking access to a computer or internet, disinterest or low digital competence.

#### Patient perceptions

During interviews, patients described that their GPs and MAs provided education, precise treatment and behavioral instructions, and detailed information about compression therapy. They considered compression therapy as very important, but some described individual barriers for its implementation and their own active participation in treatment such as pain, aesthetics and a lack of patience. Some patients mentioned that they were informed by their GPs about the possibility of participating in an e-learning, yet did not engage with it. They indicated a high relevance of verbal and practical education and described it as more important than reading information in e-learning and printed information leaflets, which were seen as potentially useful add-ons. Information on the content of the clinical picture, dressing changes, compression therapy and general measures were considered to be relevant.

*[…] I looked at the pictures a bit*,* but she [the MA] explained it all to me so well in pictures and in practice that I didn’t actually need to read it. (PAT03)*.

### Perceived effects

#### Practice team perceptions

GPs and MAs were asked to characterize their assessment of intervention program effects in both parts of the evaluation. Table 2 details their corresponding rating of perceived effects on the survey.

**Table 2:** Rating of perceived effects by GPs and MAs (*n* = 26)

Perceived effects	Median rating* (IQR)
I feel more competent now in the treatment of VLU patients.	5 (1)
My knowledge regarding local wound treatment was strengthened.	5 (1)
My knowledge regarding compression therapy was strengthened.	5 (1)
The role of the MA as case manager was strengthened.	5 (1)
Standardizing of treatment processes was improved.	5 (1)
The interventions contributed to improvements in patient care.	4 (1)
Patient education was improved.	4 (1)
Active patient participation in the treatment process was improved.	4 (1)
Software-supported patient monitoring improved the treatment process.	4 (2)

*Scale: 1 = totally disagree − 5 = totally agree

The benefits of the educational formats were considered high. Interview participants saw a decisive learning effect in content visualization and repetition in the e-learning, and a good opportunity to receive reassurance regarding compression because they had been “not entirely sure whether it always makes sense” (GP04). The flexibility in terms of being able to choose time and place of completing the training was perceived as positive. Particularly the quality of preparation of the evidence-based content, the opportunity to acquire knowledge and the strengthening of the MAs’ independent action were positively emphasized.

*So*,* with this e-learning*,* it’s quite good*,* also with the videos that you always have there*,* you perceive it differently than if you just read it through*,* at least that’s how I feel and yes*,* it’s just*,* it’s repetitive*,* but it’s good that you hear it again and again. (MA04)*.

MAs perceived the structuring of the treatment process, clarity of the documentation, and a reduction in workload as advantages of the UCC case management program. Most MAs found the monitoring function useful for documenting the healing process and enabling a comparison between the individual study visits.

*Well*,* after the patient had been here*,* we just went to the computer and entered it [the data]. I thought that […] was well guided*,* so it was actually good for the process. So*,* you could easily enter it. […] (MA10)*

GPs indicated that they had gained more confidence in caring for affected patients and ‘take over completely ourselves’ (GP04) instead of delegating the care to a specialized wound manager. Several providers felt that the intervention components had increased their overall competence in care and counseling of VLU patients, improved healing outcomes and reduced wound healing time. They also described several changes in the therapeutic approach to VLU treatment and attributed the changes to an increase in knowledge. Some stated that they had not given sufficient importance to compression therapy and had tended to focus on local wound treatment prior to their participation in UCC. Some mentioned that with the newly gained knowledge and confidence, they now applied compression therapy with greater consistency and perseverance and had learned it was worthwhile to remain patient. They expressed they were convinced that the intervention components enabled them to approach VLU care in a more structured and evidence-based manner.

*The treatment is more stringent and you reflect more on what you are doing. (GP07)*

*Yes*,* and I have to say that the wounds heal really quickly*,* well*,* from my previous time in another practice*,* there were also many older people*,* who had large wounds that almost never healed at all without compression. So that has already shown me a difference. (MA05)*

It was also stated that the role of the MA was strengthened, treatment processes were standardized and patients could be more actively integrated into the treatment process. GPs described that they readily delegated certain tasks to reduce their workload and motivate their MAs at the same time by giving them more responsibility. MAs described experiencing more joy in their profession and recognition by carrying more responsibility.

#### Patient perceptions

Patients were asked to assess potential effects they might have perceived in the context of participation in the UCC intervention. Table 3 describes their rating of perceived effects as indicated in the survey.

**Table 3:** Patient perceptions regarding effects (*n* = 11)

Item	Median rating* (IQR)
Outpatient treatment quality has improved.	5 (1)
I feel well informed about my wound now.	5 (0)
I participate more actively in my treatment now.	5 (0)
I learned to apply a compression bandage correctly.	4 (2)
I have learned how to change a dressing by myself.	5 (0)
I feel more confident in dealing with my wound.	5 (1)
I feel better treated and supported by my GP.	5 (1)

*Scale: 1 = totally disagree − 5 = totally agree

In the interviews, most patients described their experience of a positive development in their wound treatment provided by their GP practice since participating in the UCC intervention program. They felt positive about the more intensive care provided by the practice team, well informed, better treated and recognized than before. Several patients mentioned they paid more attention to a balanced diet as a result of the educational counselling of the practice team. It was also reported that the supervising MA provided knowledge about the use of compression therapy, different compression materials, padding and wound cleaning. All patients stated that they felt safer overall since participating in the study in dealing with their wound and well informed. Some patients described they were able to apply the compression bandage themselves or with the support of relatives.

*Exactly*,* we practiced it two or three times*,* when it was bandaged*,* she [the MA] said ‘now you try putting the bandage on’*,* to see if I’m doing everything right and then she gave me some help the first time. And the second time*,* it worked really well on my own. When she saw that I could do it all by myself*,* without her help*,* she knew it would work. (PAT03)*

### Improvement potential

#### Practice team perceptions

In the survey, GPs and MAs were asked to indicate whether they perceived the intervention components as time consuming. Overall, the ratings were high (meaning time-consuming), in particular regarding time required to complete the e-learning, and for patient education (med 3.4 out of 5 each), with a slightly lower rating for using standard operating procedures and the software-supported patient monitoring (med 3.2 out of 5 each). Improvement suggestions for the case management software, the e-learning and online training were mentioned neither in the interviews nor in the questionnaires. Table 4 details the combined GP and MA rating for contentedness with the intervention components.

**Table 4:** GP and MA rating of contentedness with the UCC intervention components (*n* = 26)

Intervention component	Median rating (IQR)
Synchronous online training (webinar)	5 (1)
Standard operating procedures	5 (1)
E-learning modules	5 (1)
Software-supported patient monitoring	4 (1)
Patient education material	4 (1)

*scale: 1 = totally disagree to 5 = totally agree

A comprehensive use of the UCC intervention components was considered sensible by the participants in the interview study, particularly in view of the ageing patient population and thus a potentially increasing number of wounds to be treated. In this context, a need to train social care center staff accordingly was mentioned. The willingness of patients with VLU to actively participate in a structured care program was considered likely.

#### Patient perceptions

All patients indicated in the survey that they were very content with the treatment of their wound since they had started participation in the UCC intervention, and perceived compression therapy as necessary to support wound healing (med 5 each). They also stated satisfaction with the received information material and its scope (med 4). Usefulness of the printed educational material was rated as high (med 5), and for the e-learning rather low (med 2). Time consumption for going through the educational material was perceived as moderate (med 3).

## Discussion

Findings of this process evaluation indicate a good level of adherence to the intervention and satisfaction with the UCC intervention components in all participant groups, confirming earlier findings from the UCC pilot study [22]. With the exception of the optional patient e-learning, the UCC intervention components were perceived as supportive of structured, evidence-based VLU care, facilitated gains in relevant knowledge among practice teams and patients, and simultaneously enabled informed and active participation of patients and relatives as intended by the program. A change in treatment routine towards the regular use of compression therapy as the most effective treatment measure was noted by both, practice teams and patients. Overall, the UCC approach and interventions were perceived as contributing to improvements in VLU patient care. Practice teams and patients alike positively perceived the strengthened and central role of MAs as case managers and their increased involvement in wound treatment and patient education. Overall, these findings are largely consistent with expectations from a pre-intervention survey study [25], findings of the process evaluation in the UCC pilot implementation [22], and the outcome analysis of the UCC trial [23].

The UCC program intends to enhance knowledge and practical skills of care providers, and promote informed and active participation of patients to support standardized evidence-based and patient-centered treatment of VLU in GP practices. Implementation of evidence-based interventions in care settings requires behavioral changes in patients and care providers. Frameworks such as the Theoretical Domains Framework (TDF) can support reflection of the complexity of influencing factors, both in the care organisation and in individuals [49, 50]. The process evaluation conducted within the UCC pilot study for instance used the TDF as analytical framework to inform intervention refinement and meet identified challenges and needs. One of the identified challenges referred to the patient e-learning which was used by only one patient in the pilot study due to a lack of digital affinity, technical equipment, and support from relatives [22]. Since a reduction of accessibility barriers for older patients and alternative formats other than the printed information material were outside the scope of the study center, the intervention was adapted for the RCT to meet this challenge and the patient e-learning then was offered as optional component in combination with the printed patient information. Prior to a broader implementation of the UCC intervention in older primary care populations, a future program adaptation could thus consider development of blended learning components and on-site educational formats to accommodate for patient preferences and needs.

The transfer of knowledge via the on-demand online format offered great flexibility for practice teams in terms of both, organization and scheduling. The data collected in this process evaluation show that the practice teams gained relevant knowledge by participating in the training offered, and that this increase in competence was reflected in perceived treatment successes. In this context, a rethinking of the role of GP practices towards active treatment of VLU patients as primary care providers and a change in attitude towards compression therapy was also observed. A focus shift from purely local wound treatment to causally oriented treatment with regular application of guideline-compliant compression therapy was also identified. Previous research indicated knowledge deficits regarding compression therapy and its practical application among healthcare professionals [12, 51–53]. However, it is considered to be the most effective conservative VLU treatment option with the potential of halving healing time when adequately applied [54, 55]. Research also points to knowledge gaps, pain and discomfort, and socio-psychological patient factors as determinants for patient adherence to compression therapy [56–59]. Educational interventions can improve patient knowledge and close such gaps, thus improving therapy adherence and wound-related as well as patient-reported outcomes [29, 60–64]. As intended, the targeted training and educational interventions offered in the UCC program supported patients in taking a more active role in the treatment process and overcoming barriers together with their primary care providers. With regard to a broader implementation of the UCC intervention program, the knowledge transfer format used for practice teams can thus be recommended since it successfully addressed potential knowledge gaps. For older patients in particular, the on-demand e-learning format offered for voluntary use in the UCC program might be less relevant than engagement in verbal and practical education supported by printed information material. This is supported by the strong patient activation observed in this process evaluation, despite the only sporadic use of the patient e-learning. However, the patient e-learning could be a suitable add-on for patients interested in this format. Follow-up research should therefore include and evaluate this option.

In light of scarce resources and an expected increase in multimorbidity and chronic wounds due to aging populations, the strengthening and expansion of the MA role combined with a structured case management approach as used in the UCC program seems to be essential to ensure adequate VLU care in general practice in the future. This approach was strongly advocated by all participants in this process evaluation and is supported by research that indicates that a trustful relationship between nursing staff and patients can foster therapy adherence [65, 66].

### Strengths and limitations

Studies on care provider and patient perspectives regarding a holistic case management program for VLU in general practice are scarce. The methodological approach in this process evaluation facilitated in-depth analysis of intervention acceptance and perceived effects, both by general practice teams and patients. Conducting the interviews via telephone accommodated for practice team and patient limitations regarding time resources and potential burden. Participants were able to comprehensively describe their perceptions regarding the program and perceived effects. The systematic yet flexible approach to structuring the qualitative data enabled cross-category comparisons, identification of key aspects and potential contrasts. The first author, who was not involved in data collection, analyzed all qualitative data based on initial analysis of *n* = 15 transcripts by a junior researcher and along consensus processes in the research team, thus ensuring a high degree of objectivity and independence. With a sample size of *n* = 27 interviews, a high degree of information saturation was achieved [67]. The convergent mixed-methods approach allowed for integration of survey and qualitative data to identify common concepts across both data sources. Reporting follows scientifically valid criteria [68].

Some limitations have to be reported. Statements regarding the intervention program might entail a limited transferability since less motivated practice teams could have reported different perceptions. Process evaluation data were collected in the intervention group only and participation was voluntary. Participants received a small financial compensation for taking part in the survey and/or an interview and it cannot be excluded that this influenced their response patterns in the survey. However, the statements in the interviews supported the ratings letting such an influence appear to be rather unlikely. Since gaining insights into necessary component refinement was not a goal for this process evaluation, no implementation research framework was used as an analytic framework.

The patient e-learning was an optional intervention component and no statements could be collected regarding a perceived effect or improvement potential. Transcripts and results were not returned to participants. The use of non-validated study-specific questionnaires with unclear psychometric properties and an incomplete response rate might limit the validity and reliability of the survey data. Ceiling effects and discriminability could not be accounted for. In both parts of the study, the targeted sample size could not be reached. Overconfidence and social desirability bias may have affected generalizability of the results of the survey and interviews alike.

## Conclusions

Findings of this process evaluation indicate that the UCC intervention program facilitated a relevant gain in knowledge for practice teams and patients, promoted active patient participation, and a shift away from simple wound dressing changes towards a comprehensive treatment with regular application of compression therapy. The program can be considered suitable for a broader disease management approach. The outcome analysis in the UCC trial confirmed the perceived effects [23].

## Electronic Supplementary Material

Below is the link to the electronic supplementary material.

Supplementary Material 1

Supplementary Material 2

## Data Availability

All data generated and analyzed in this process evaluation are stored on a secure server at the Department of General Practice and Health Services Research, University Hospital Heidelberg, Germany. De-identified sets of these data can be made available by the corresponding author on reasonable request.

## Abbreviations

GP: General practitioner
MA: non-physician assistant (medical assistant)
SOP: Standard operating procedures
UCC: Ulcus Cruris care
VLU: Venous leg ulcer
