# Supplementary material for: Implementing a primary care disease management concept for venous leg ulceration: findings of a mixed-methods process evaluation in the Ulcus Cruris Care trial
Source: BMC Health Serv Res. 2026 May 7;26:664. doi: 10.1186/s12913-026-14674-0 (PMC13154889; doi:10.1186/s12913-026-14674-0)
Supplement: Supplementary file 1 — Supplementary Material 1 [file 12913_2026_14674_MOESM1_ESM.docx]

**Additional file 1:**

**Process evalaution in the Ulcus Cruris Care project - survey topics**

**Practice teams**

Items referring to **Intervention fidelity:**

The practice used (yes/no)

the software-supported monitoring
the Standard Operating Procedures. If so, did you use them regarding all intervention patients?
the patient e-Learning as patient information? If so, for all intervention patients?
the printed patient information material? If so, for all intervention patients?

If not: Please provide reasons (free text fields)

Relevance of educational content (check where applicable)

Webinar: pathophysiology, compression therapy, local wound treatment, patient information and promotion of active participation in the treatment process

E-learning for practice teams: pathophysiology, compression therapy, local wound treatment, patient information and promotion of active participation in the treatment process

Items referring to **Perceived effects** (scale: Totally disagree - Totally agree)

My knowledge about compression therapy was promoted.
My knowledge about local wound treatment was promoted.
Since participation in this study I feel more competent in VLU care.
The role of the non-physician medical assistant/case manager got strengthened.
The software-supported patient monitoring improved the treatment process.
Standardization of the treatment process improved.
Patient information and education improved.
Active patient participation in the treatment process improved.
The interventions contributed to improvement of patient care.

| **Unexpected positive effects:** |
| --- |

(free text field)

| **Unexpected negative effects:** |
| --- |

(free text field)

Items referring to **Improvement potential:**

It took up a lot of time to (scale: Totally disagree - Totally agree)

complete the e–learning modules
apply the Standard Operating Procedures
use the software-supported patient monitoring
inform and educate patients

I am content with the (scale: Totally disagree - Totally agree)

Webinar
E – learning modules
Standard Operating procedures
Software-supported patient monitoring
Patient e-learning and printed patient information material

If not: Please provide reasons (free text fields)

Intervention components are applicable for VLU care (scale: Totally disagree - Totally agree)

Online educational webinar
E – learning modules
Standard Operating procedures
Software-supported patient monitoring
Patient e-learning
Printed patient information material

Suggestions for improvements (free text fields)

**Patients**

**Intervention components:** (yes/no)

The general practice team explained my disease and wound treatment at the start of the study.
I was informed about usefulness and effect of compression therapy.
I was informed about correct, independent wound dressing change.
I received recommendations for vein exercises and physical activity.
I received printed information material.
I read the printed information material.
I was informed about the optional patient e-learning.
I used the patient e-learning modules.

If not: Please provide reasons (free text fields

Usefulness: (scale: Totally disagree - Totally agree)
Patient e-learning
printed information material

Relevance of content: (scale: Totally disagree - Totally agree)

Patient e-learning: pathophysiology, compression therapy, local wound treatment, wound dressing change, physical activity, general measures (skin and body care, diet, compression devices)

Information material: pathophysiology, compression therapy, local wound treatment, wound dressing change, physical activity, general measures (skin and body care, diet, compression devices)

**Perceived effects** of participation in the study: (scale: Totally disagree - Totally agree)

The quality of wound care improved through study participation.
I feel well informed.
I participate more actively in the treatment process.
I learned how to apply a compression bandaging correctly and what to pay attention to in doing so.I learned how to change a bandage independently and correctly.
I feel more secure about dealing with my wound now.
I feel better treated and more supported by my general practitioner.
I am content with the treatment of my wound since the start of the study.

**Improvement potential:**

It took up a lot of time to (scale: Do not agree at all - Totally agree)

read the information material
complete the e-learning modules

I am content with the (scale: Totally disagree - Totally agree)

patient e-learning
printed information material

If not: Please provide reasons (free text fields)

The intervention components were applicable. (scale: Totally disagree - Totally agree)

Patient e-learning
Printed patient information material

Suggestions for improvements (free text fields)
