# Supplementary material for: Implementing a primary care disease management concept for venous leg ulceration: findings of a mixed-methods process evaluation in the Ulcus Cruris Care trial
Source: BMC Health Serv Res. 2026 May 7;26:664. doi: 10.1186/s12913-026-14674-0 (PMC13154889; doi:10.1186/s12913-026-14674-0)
Supplement: Supplementary file 2 — Supplementary Material 2 [file 12913_2026_14674_MOESM2_ESM.docx]

**Additional file 2**

**Process evalaution in the Ulcus Cruris Care project - interview topics**

**Practice teams:**

*Intervention components*

- implementation into VLU care of
- educational contents
- standard operating procedures
- software-supported case management and patient monitoring
- patient education
- perceived benefits
- potential challenges

*Intervention program*

- general acceptance of program
- perceived effects of intervention program
- perceived changes in provided care
- perception regarding transferability
- potential for optimization

**Patients:**

- wound care
- disease-related information
- information material and e-learning
- treatment
- case management and role of non-physician assistant
- perceived benefits and effects
- challenges
- potential for optimization
